# Comparison of Full-Field Stimulus Threshold Measurements in Patients With Retinitis Pigmentosa and Healthy Subjects With Dilated and Nondilated Pupil

**DOI:** 10.1167/tvst.13.4.23

**Published:** 2024-04-17

**Authors:** Milda Reith, Katarina Stingl, Laura Kühlewein, Melanie Kempf, Krunoslav Stingl, Hana Langrova

**Affiliations:** 1 University Eye Hospital, Center for Ophthalmology, University of Tübingen, Tübingen, Germany; 2 Center for Rare Eye Diseases, University of Tübingen, Tübingen, Germany; 3 Charles University, Medical Faculty in Hradec Kralove and Faculty Hospital, Department of Ophthalmology, Czech Republic

**Keywords:** full-field stimulus testing, retinitis pigmentosa, pupil dilation

## Abstract

**Purpose:**

The common protocol of full-field stimulus threshold (FST) testing recommends pupil dilation. The aim of this study is to investigate the difference between FST measurements with dilated and nondilated pupils in healthy subjects and patients with retinitis pigmentosa (RP).

**Methods:**

Twenty healthy subjects and 20 RP patients were selected. One pupil of each subject was dilated; the other eye was measured in physiological width of the pupil. The FST was conducted using Diagnosys Espion E2/E3 with white, blue, and red stimuli. Statistical analysis was conducted with a mixed-model analysis of variance and a paired *t*-test.

**Results:**

The statistical analysis revealed a significant difference between measurements of dilated and nondilated pupils with the following: blue stimuli for all subjects and groups except those with highly progressed RP; white stimuli for all tested subjects in total, for RP patients with better-preserved visual field (VF), and rod-mediated FST response; and red stimuli for RP patients with better-preserved VF and rod-mediated FST response. On average, the difference between the FST values for RP patients were −3.2 ± 3 dB for blue, −2.3 ± 2.9 dB for white, and −0.83 ± 3 dB for red stimuli. The correlation between the FST values of dilated and nondilated pupils with all three stimuli was linear.

**Conclusions:**

Current recommendations are to perform FST with dilated pupils. However, based on this study's findings, pupil dilation can be omitted for clinical diagnostics or rough follow-ups.

**Translational Relevance:**

Our data provide useful information for the clinical use of FST.

## Introduction

Full-field stimulus threshold (FST) testing is a psychophysical test that has been established as an important readout for functional rod rescue after genetic therapies for inherited retinal diseases (IRD).^1^^–^^6^ In particular, the chromatic FST response has been emphasized as a unique characteristic of this tool because it enables determination of whether the dark-adapted thresholds are mediated by cones or rods. Physiological rod-mediated thresholds with blue and red light have a difference of 25 dB, whereas this difference is below 10 dB for cone-mediated thresholds.^1^^,^^4^^,^^7^^,^^8^

FST has been shown to correlate with other clinical diagnostics, including visual acuity, macular thickness, the electroretinogram in Stargardt patients,^9^ optical coherence tomography (OCT), and the hyperfluorescent ring of fundus autofluorescence in retinitis pigmentosa (RP) patients,^10^ as well as the duration of the disease.^11^ Additionally, in cases other than IRD, attempts have been made to use FST as a diagnostic tool.^12^ A survey of RP patients with RPE-65 mutations showed that FST is used to evaluate these patients in 36% of centers in Europe.^13^

Regular use of FST requires consideration of the feasibility of the testing algorithm and possible interferences. The current algorithm of FST testing requires the dilation of the pupils. To our knowledge, however, this requirement has not been tested in a clinical setting. Therefore the aim of this study is to investigate the differences between FST testing with dilated and nondilated pupils and to improve the understanding of the effect of pupil size on FST measurements.

## Material and Methods

This study was approved by the Ethics Committee of the University Hospital of Tuebingen, and informed written consent was obtained from all subjects. The procedures were conducted in accordance with the Declaration of Helsinki. In total, 20 RP patients and 20 healthy subjects were recruited at the University of Tuebingen, Germany.

The RP patients were selected according to the findings of the visual field (VF). Ten patients displayed a progressed stage of the disease, with a central VF of approximately 10° to 20°. The other 10 patients showed a better-preserved VF with either larger remaining central areas or annular scotomas. All patients underwent a complete ophthalmological investigation, including best-corrected visual acuity (BCVA), kinetic perimetry using Octopus 900 (Haag-Streit, Wedel, Germany) with the III4e stimulus, OCT using Octopus 900 (Haag-Streit, Wedel, Germany) with the III4e stimulus, and fundoscopy in mydriasis. Patients with asymmetrical findings between the left and right eye or with relevant findings in the macula, such as macular edema, were not considered to ensure comparison between eyes was possible.

For the FST, one pupil of each subject was dilated (tropicamide 5 mg, phenylephrine-HCl 25 mg); the other eye of the same patient was measured in physiological width (miosis). The order of testing—that is, the dilated or non-dilated eye—was randomized. The subjects were required to dark-adapt for at least 30 minutes. The testing was conducted using Diagnosys Espion E2/E3 (Diagnosys LLC, Cambridge, UK) using white, blue, and red stimuli with 0 dB set to 0.01 cd/m^2^. Data are presented as mean values ± standard deviation.

The statistical analysis was conducted using a mixed-model analysis of variance with factor groups (healthy subjects and RP patients) and dilatation (dilated and nondilated pupils). Subgroup analysis in RP patients was performed using a paired *t*-test. The dependency between FST values from dilated and nondilated pupils’ measurements was tested with linear regression. The statistical tests were performed in MATLAB or MS Excel.

## Results

### Subjects’ Characteristics

The group of healthy subjects included 14 female and six male subjects. The median age was 36 years, and the BCVA was between 0.0 and −0.1 logMAR. The group of RP patients comprised nine females and 11 males, and the median age of this group was 45 years. BCVA was between “hand motion” and 0.0 logMar. The included patients were diagnosed with either autosomal recessive, autosomal dominant, x-chromosomal, or syndromal RP. The median duration of the disease was 21.5 years, and the genotype was known in the majority of the cases from the medical history. Previously conducted electroretinograms performed according to ISCEV standards were also available for most cases. The detailed characteristics are shown in Table 1.

**Table 1.** Summary of RP Patients’ Characteristics.

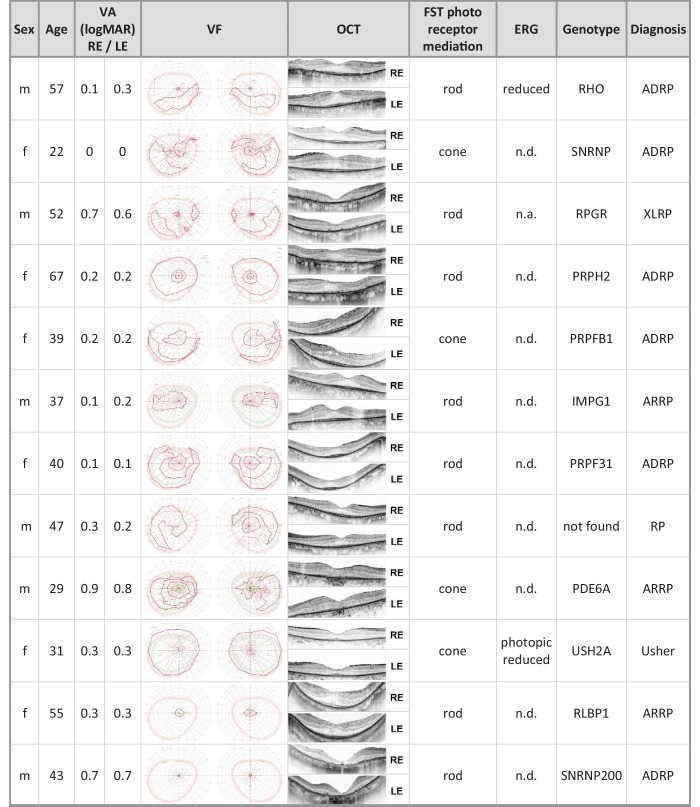
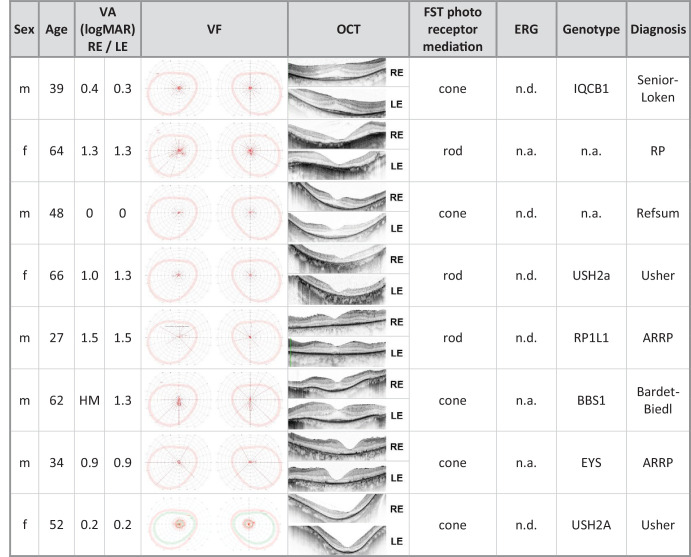

ADRP, autosomal dominant retinitis pigmentosa; ARRP, autosomal recessive retinitis pigmentosa; F, female; HM, hand movement; LE, left eye; M, male; n.a., not available; n.d., not detectable; RE, right eye; VA, visual acuity; XLRP, X-linked retinitis pigmentosa.

### The Difference of FST Values for Measurements With Dilated and Nondilated Pupils

The *p* values of the differences between FST measurements with dilated and nondilated pupils in different tested groups are shown in the Table 2 and described below.

**Table 2.** *P* Values of the Difference Between FST Values if Measured With Dilated or Nondilated Pupils for the Different Chromatic and White FST Stimuli

		FST Stimuli		
Subject Group	n	Blue	Red	White
All subjects	40	0.05^*^	0.08	0.04^*^
RP patients	20	0.02^*^	0.17	0.44
RP VF < 10°–20°	10	0.05^*^	0.36	0.10
RP VF > 10°–20°	10	0.02^*^	0.04^*^	0.002^*^
RP rod-mediated	12	0.002^*^	0.008^*^	0.02^*^
RP cone-mediated	8	0.07	0.14	0.31

FST, full-field stimulus testing; n, sample size; RP, retinitis pigmentosa; VF, visual field.

* *P* ≤ 0.05.

#### All Subjects

**Figure 1.**
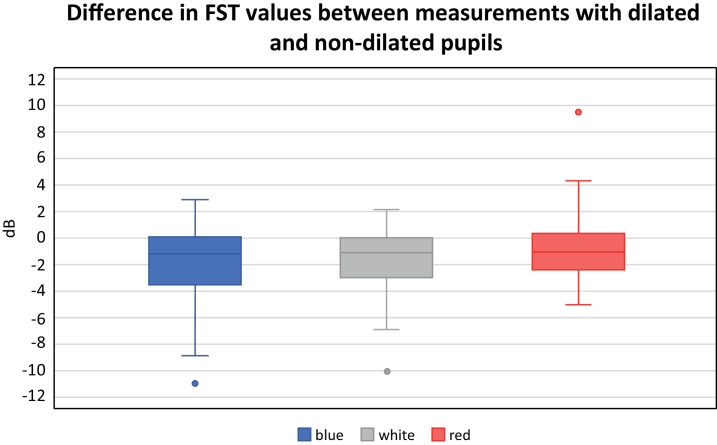
The difference in FST values between measurements with dilated and non-dilated pupils using blue, white, and red stimuli in dB of all tested subjects.

The FST results for white and blue stimuli in all tested subjects showed a statistically significant difference between the measurements with dilated and nondilated pupils (*P* = 0.04; *P* = 0.05, respectively). However, only a statistical trend was found for the red stimulus (*P* = 0.08). On average, the difference between the FST values was −2 ± 3.0 dB for blue; −1.8 ± 2.6 dB for white, and −0.7 ± 2.5 dB for red stimuli (Fig. 1).

#### RP Patients

**Figure 2.**
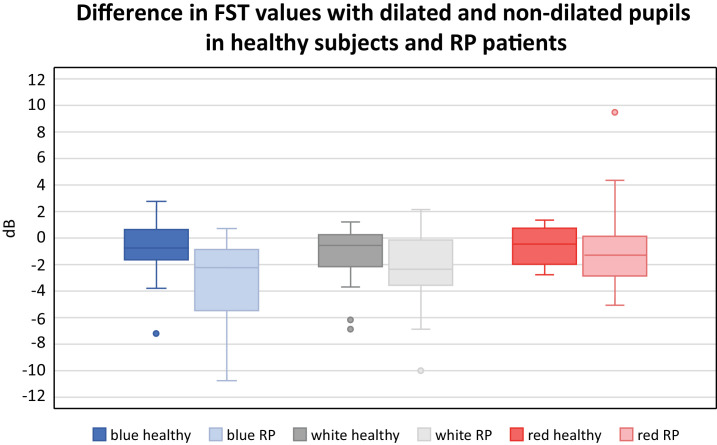
The difference in FST values between measurements with dilated and non-dilated pupils in dB using blue, white, and red stimuli by group: healthy subjects or RP patients.

For RP patients, a statistically significant difference was observed between measurements with dilated or nondilated pupils when measured with blue stimuli (*P* = 0.02), but no statistical significance was found for white (*P* = 0.44) or red stimuli (*P* = 0.17). On average, the difference between the FST values was −3.2 ± 3.1 dB for blue, −2.3 ± 2.9 dB for white, and −0.8 ± 3.2 dB for red stimuli. The distribution of the differences for all three stimuli in healthy subjects and RP patients is shown in Figure 2.

#### A Selected Example of FST Values for Blue and Red Stimuli in one Healthy Subject and one RP Patient

**Figure 3.**
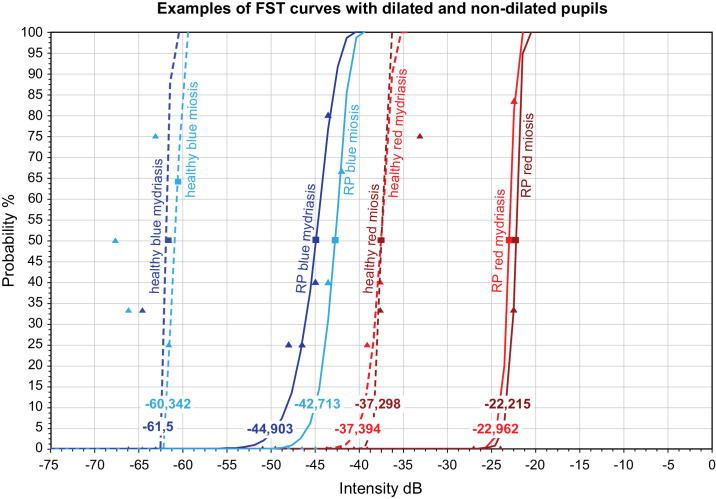
Example of the obtained FST curves for one healthy subject and one RP patient for red and blue stimuli with dilated and non-dilated pupils.

The FST curves of one healthy subject and one RP patient for red and blue stimuli with dilated and nondilated pupils are depicted in Figure 3 as an example. The FST values in a healthy subject with dilated pupils were −61.5 dB for blue and 37.4 dB for red stimuli, and −60.3 dB and −37.3 dB, respectively, with nondilated pupil. In the RP patient, the FST values with pupil dilation were −44.9 dB for blue and 23.0 dB for red stimulus, while they were −42.7 dB and −22.2 dB, respectively, without pupil dilation.

#### Subanalysis of the FST Values in RP Patients According to the VF

In the subgroup of RP patients with advanced constriction of the VF, FST values with blue stimuli were significantly different if measured with or without pupil dilatation (*P* = 0.05), but no difference was found with white or red stimuli (*P* = 0.1; *P* = 0.361, respectively). The difference in FST values was, on average, −4.3 ± 3.4 dB for blue, −2.6 ± 3.9 for white, and −1.3 ± 2.7 dB for red stimuli.

In the subgroup analysis for RP patients with better-preserved VF, the FST results showed statistically significant differences if measured with or without pupil dilatation for white (*P* = 0.002), red (*P* = 0.04), and blue (*P* = 0.02) stimuli. The difference was −2.3 ± 2.9 dB for blue, −2.2 ± 1.8 dB for white, and −2.1 ± 1.9 dB for red stimuli.

#### Subanalysis of the FST Values for RP Patients According to the Predominant Photoreceptor Mediation

In the subgroup with the cone-mediated response, no statistically significant difference was found between FST values with blue, white, or red stimuli when measured with dilated or non-dilated pupils (*P* = 0.07; *P* = 0.14; *P* = 0.31, respectively). The average differences were 2.8 ± 3.4 dB for blue, −1.4 ± 2.3 dB for white, and 1.0 ± 2.6 dB for red stimuli.

However, in the subgroup of RP patients with rod-mediated responses, a statistically significant difference was observed between measured values with blue (*P* = 0.002), white (*P* = 0.008) and red (*P* = 0.02) stimuli. The average responses were elevated by −4.00 ± 3.4 dB for blue stimuli, by −3.0 ± 3.2 dB for white stimuli, and by −1.9 ± 2.3 dB for red stimuli.

#### The Relationship of Pupil Dilation and FST Values in Healthy Subjects and RP Patients

**Figure 4.**
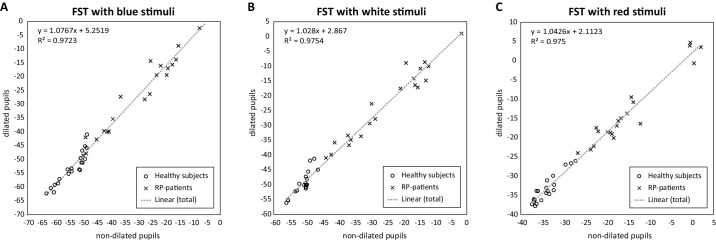
The values of FST measurements (dB) with blue (**A**), white (**B**), and red (**C**) stimuli in healthy subjects (circles) and RP patients (x) with dilated and nondilated pupils.

We explored the relationship between FST values in dilated and non-dilated measurements using linear regression with *r*^2^ = 0.97 for blue, *r*^2^ = 0.98 for white, and *r*^2^ = 0.98 for red (Fig. 4). In summary, we showed a statistically significant difference between FST measurements with dilated and nondilated pupils with blue stimuli for all tested subjects and subgroups, except the most-advanced RP cases with cone-mediated FST response. For white stimuli, statistical significance was found when calculated for the entirety of the tested subjects, as well as RP patients with better-preserved VF and rod-mediated FST responses. For red stimuli, statistical significance was shown for RP patients with better-preserved VF and rod-mediated FST responses.

## Discussion

FST was developed as an improved, full-field version of dark adaptometry and became the most commonly used readout for rod function. Its increased standardization as a commercial test allows widened use at different clinical sites. The possibility of detecting and thus measuring the function of severely but not homogeneously affected retinas is one of the reasons this test has gained significance. However, the practical perspective is not to be underestimated. Conducting FST requires dark adaptation (at least 30 minutes), as well as an experienced and motivated technician. Nevertheless, the test is more comfortable for patients than traditional electrophysiology, and after dark adaptation is completed, a single test takes only several minutes. Thus, in a clinical setting with an experienced technician, the test can be acquired over a reasonable period of time. Because of acceptable retest variability,^14^ FST values using blue and red light appear to be good progression markers of rod and cone degeneration after electroretinographic signals are no longer detectable. In clinical trials, however, repetition of FST measurements is frequently required for higher reliability of values. This can be tedious for the patient, regardless of the pupil size. Therefore, for routine FST measurement, understanding that the examination has value even without pupil dilation might increase the willingness of patients, especially children, to undergo the test.

Pupil dilation might affect how much of the retinal surface can be illuminated by the stimulus and thereby cause a difference in the measured threshold. The origin of the FST has been shown to originate from the most sensitive region of the retina,^14^ which is located in the central 20° in RP patients.^15^ In choroideremia, however, the FST response has been shown to include not only the response of the most sensitive region but also, to some extent, a summation of the peripheral regions.^16^

Our results showed the effect of pupil dilation on the FST not only in RP patients but also in healthy subjects. Therefore the effect of pupil dilation seems to have a general impact independent of the functional state of the retina. Assuming reduced illumination of the retina through a miotic pupil, both previously mentioned factors could play a role: exclusion of the most sensitive surviving photoreceptors (if they are located more peripherally in RP patients) or reduction of the role of spatial summation through the exclusion of retinal regions in healthy subjects.

We conducted a subanalysis of RP patients with different levels of severity based upon VF constriction and the different photoreceptor mediation of the FST response. The results for the group with progressed VF constriction showed a statistically significant difference only with blue stimuli. For the group with better-preserved VF, significant differences were found with all three stimuli. Although VF expresses the progress of the IRD in clinical terms, it has been reported as correlating poorly with FST.^15^

In RP patients, photoreceptor mediation has been shown to shift from rod to cone mediation over the natural course of the disease.^14^ Our data for the group with cone-mediated FST-response showed no statistically significant differences with either of the stimuli, whereas statistically significant differences with all the stimuli were observed for the subjects with rod-mediated FST-response. Presumably, the effect of pupil dilation seems to be less relevant at the end stage of RP.

The FST measurements with blue stimuli showed consistent results throughout all analysis groups, with the exception of the subjects with cone-mediated FST responses. This was consistent with the claim that rods are more sensitive to blue than to red; cones are equally sensitive to both.^17^ The fact that FST measurements with nondilated pupils in healthy individuals showed a difference when measured with blue and white stimuli supports the assumption that the blue stimulus is more sensitive to the pupilar width of the chromatic stimuli.

The mean difference of the measurements of dilated and non-dilated pupils for FST with blue stimuli in our study was −2 dB. As a psychophysical test, FST is subject to fluctuations and is dependent on many variables.^1^ The inter-session repeatability of FST has been reported by a coefficient of ± 2.3–2.7 dB for chromatic FST^16^ and ±3.9 dB for white stimuli,^14^ but this may vary between different testing sites. Accordingly, the mean difference of an FST value in dilated versus non-dilated pupils is lower than the test-retest variability of the test. Although the differences, especially when testing with the blue stimulus, were statistically significant for almost all groups, the actual difference of 2 to 3 dB is of limited clinical significance.

Clinical trials with gene therapy, especially for voretigene neparvovec, have shown a clear decrease of the thresholds, initially by 18 to 45 dB.^3^^,^^5^^,^^18^^–^^20^ Advanced RP patients often show a pronounced elevation of the FST value by an average of 41 dB.^21^ Early cone-rod dystrophies, however, have been reported as showing a near-normal FST response.^14^^,^^21^ For some questions, such as detecting progression of IRDs,^8^^,^^11^ regular follow-ups,^22^ and treatment assessments,^4^ much subtler changes of the FST ranging from 0.5–7.8 dB can be of great relevance; however, changes in the follow-up that are below the test-retest variability are challenging to decipher. Post-treatment (voretigene neparvovec) follow-ups were shown to have various amounts of fluctuation depending on the initial change of the rod sensitivity, thus showing the necessity of an exact measurement of the threshold.^6^

In summary, FST should ideally be conducted with dilated pupils, and if evaluating treatment effects, the same condition must be guaranteed before and after treatment. However, in routine clinical practice, FST can be performed with nondilated pupils if only diagnostics are required. This is especially important in the routine diagnostics of children or patients who refuse pupil dilatation, as is the case for many IRD patients due to their pronounced photophobia. The linearity of the data allows a good level of prediction of the FST values if the test was conducted with nondilated pupils with correlation coefficients > 0.9.

This study did face some limitations. First, precise measurements of the pupil size were not conducted; the results claim only the effect of assumed pharmacological mydriasis and physiological miosis. The duration of the dark adaptation has been shown to have an impact on rod sensitivity^1^ and may also affect the pupil width in healthy individuals and RP patients in different ways. Moreover, the small sample sizes, especially in the subanalysis, must be considered. Further effects of age or the pharmaceutical effects of dilating eye drops cannot be ruled out. Nevertheless, the setup of this study complies with clinical practice.
